# Health in All Policies in Sectoral Legislation: A Content Analysis of Selected Laws in the Republic of Srpska

**DOI:** 10.3390/healthcare14142139

**Published:** 2026-07-16

**Authors:** Biljana Mijović, Bojan Joksimović, Milena Dubravac Tanasković, Jovan Kulić, Zorica Stojanović, Nataša Stojić, Ljiljana Milošević, Biljana Panin, Galina Ćurčić, Dunja Prokić, Jelena Djekic Malbasa, Predrag Duric

**Affiliations:** 1 Faculty of Medicine Foča, University of East Sarajevo, 73300 Foča, Bosnia and Herzegovina; biljana.mijovic@ues.rs.ba (B.M.); bojannjoksimovic@gmail.com (B.J.); menadub@gmail.com (M.D.T.); zorica.stojanovic@ues.rs.ba (Z.S.); 2 Faculty of Environmental Protection, Educons University, 21208 Sremska Kamenica, Serbia; natasa.stojic@educons.edu.rs (N.S.); ljiljana.curcic@educons.edu.rs (L.M.); biljana.panin@educons.edu.rs (B.P.); galina.curcic@educons.edu.rs (G.Ć.); dunja.prokic@educons.edu.rs (D.P.); predrag.duric@educons.edu.rs (P.D.); 3 Faculty of Medicine, University of Novi Sad, 21137 Novi Sad, Serbia; jelena.djekic-malbasa@mf.uns.ac.rs; 4 Institute for Pulmonary Diseases of Vojvodina, 21204 Sremska Kamenica, Serbia; 5 Faculty of Pharmacy Belgrade, Educons University, 11000 Belgrade, Serbia

**Keywords:** Health in All Policies, HiAP, legislative analysis, health equity, intersectoral governance, social determinants of health, legal epidemiology

## Abstract

**Background:** The Health in All Policies (HiAP) approach promotes the systematic integration of health considerations into policymaking across non-health sectors to address social determinants of health and improve population health outcomes. Although increasingly recognized internationally, the extent of its legislative implementation in the Republic of Srpska remains unclear. **Objective:** To assess the extent to which HiAP principles are embedded in sectoral legislation in the Republic of Srpska and to identify strengths and gaps in current legal frameworks. **Methods:** A structured content analysis of the text of laws was conducted across ten priority sectors. Twenty-two laws were reviewed using an analytical framework based on five core HiAP components: health outcomes, intersectoral governance, health equity, evidence-informed policymaking, and monitoring and accountability. The presence and distribution of these components were systematically examined across sectors. **Results:** Health considerations were frequently reflected in the text of the analysed legislation. Explicit references to health outcomes were identified in more than 60% of the laws, while references to intersectoral governance were identified in more than 40%. However, integration was uneven across sectors. Health equity principles were inconsistently incorporated, while monitoring, evaluation, and accountability mechanisms were insufficiently developed, with explicit provisions found in fewer than half of the laws. No analysed law formally mandated Health Impact Assessment as a policy instrument. **Conclusions:** The Republic of Srpska has an existing legislative basis for advancing HiAP, but implementation remains fragmented rather than systematic. Addressing identified gaps—particularly in health equity, Health Impact Assessment, and health-oriented monitoring mechanisms—through targeted legislative reforms could improve policy coherence, accountability, and long-term sustainability. To our knowledge, this is the first systematic content analysis of HiAP integration in the sectoral legislation of the Republic of Srpska; it applies a replicable analytical framework derived from the WHO HiAP Framework for Country Action that is transferable to other decentralised and transitional governance settings.

## 1. Introduction

The Health in All Policies (HiAP) approach represents a comprehensive framework for public governance that recognizes health as an outcome shaped not only by healthcare systems, but also by decisions taken across a wide range of non-health sectors [1]. Rooted in the social determinants of health paradigm, HiAP emphasizes that decisions in sectors such as education, employment, transport, environment, or urban development exert substantial direct and indirect effects on population health [2]. Although the principles underlying HiAP can be traced to early public health initiatives and the foundational orientations of the World Health Organization, including its 1948 Constitution and subsequent milestones such as the Declaration of Alma-Ata and the Ottawa Charter for Health Promotion, the approach emerged as a coherent policy framework in the early 21st century [3,4,5]. It gained international prominence following its endorsement at the Eighth Global Conference on Health Promotion held in Helsinki in 2013 [1,6]. Unlike earlier forms of intersectoral action for health, which often relied on time-limited partnerships addressing specific issues, HiAP represents a shift toward systematically embedding health and equity considerations within sectoral policies themselves [2]. Within this governance-oriented framework, legislation plays a critical role in operationalizing HiAP principles, as laws define sectoral mandates, regulatory standards, preventive measures, and governance arrangements that directly or indirectly influence health outcomes [2,7]. Central to HiAP are mechanisms such as intersectoral collaboration, shared accountability, capacity building, joint resourcing, and policy tools including Health Impact Assessment (HIA), which together aim to enhance policy coherence and anticipate the health and equity consequences of public decision-making [2,7].

Despite the growing recognition of the importance of HiAP, empirical evidence suggests that health considerations are often only partially or implicitly reflected in sectoral legislation, particularly in areas traditionally regarded as outside the health domain [8,9]. Establishing priorities for HiAP requires consideration of policy feasibility, power dynamics, and the broader national context. This includes strategic prioritization, systematic assessment of governance capacities and resources needed for implementation [2]. Policies governing education, agriculture, emergency management, water resources, urban and spatial planning, labour, environmental protection, transport, energy, and local self-government may substantially influence determinants of health such as safety, environmental exposures, working conditions, access to services, and resilience to emergencies, yet these effects are not always systematically assessed or explicitly addressed within legal texts [8,9]. Assessing the extent to which legislation incorporates HiAP principles is therefore important for understanding policy readiness for intersectoral action and identifying opportunities to strengthen health-oriented governance. Analysing the content of legislation in this way is consistent with the established public health research tradition of legal epidemiology and policy surveillance, in which the text of laws is treated as data and systematically coded against defined indicators [10]. A central premise of this tradition is that the wording of statutes is a legitimate and consequential object of study in its own right: the presence, framing, and specificity of statutory provisions shape the obligations, mandates, and institutional arrangements that are available for implementation, and these textual features can be characterised independently of, and prior to, any evaluation of enforcement or health outcomes [10,11]. Systematically measuring what the law says is widely regarded as a necessary first step in the policy evaluation cycle, since the content of a legal text constitutes the independent variable whose downstream effects later studies seek to estimate [11]. Examining the content of law therefore yields a baseline that documents where health is named, how it is framed, and which governance, equity, evidence, and accountability mechanisms are codified—information that is a prerequisite for, rather than a substitute for, subsequent implementation and outcome research [10,11].

This issue is particularly relevant in Bosnia and Herzegovina, a country characterized by a highly decentralized and complex governance structure, with some competencies exercised at the state level, while others are shared with, or devolved to, lower levels of government [12]. The country consists of two main entities—the Federation of Bosnia and Herzegovina and the Republic of Srpska—as well as the self-governing Brčko District [12]. Furthermore, the Federation of Bosnia and Herzegovina is internally divided into ten cantons, each possessing significant legislative and executive authority [12].

Such a multilayered institutional arrangement presents challenges for implementing cross-sectoral approaches such as HiAP, which depend on policy coherence, intersectoral coordination, and sustained collaboration across sectors and levels of government. Fragmented competencies and overlapping jurisdictions may hinder the development of a unified approach to integrating health considerations into public policies.

For these reasons, this analysis focuses on the Republic of Srpska as a single administrative and legislative unit that allows a more coherent examination of policy integration processes. This is a deliberate scoping decision rather than a claim that the entity is representative of the country as a whole. Because the entity exercises consolidated legislative competence over the sectors examined, its statutes can be coded against a common framework without the confounding introduced by the cantonal and state-level fragmentation that characterises the Federation of Bosnia and Herzegovina and the Brčko District. The trade-off is that the present findings describe one entity only and cannot be generalised to the Federation, the Brčko District, or the state level, nor do they capture the cross-entity coordination dynamics that a country-wide HiAP analysis would entail; these are addressed as limitations and as directions for the comparative work of which this study forms part. Although HiAP has not been codified through a dedicated legal framework, elements of this approach can be identified across a range of sectoral policies and governance mechanisms that recognize the shared responsibility of multiple sectors in promoting population health and health equity. Influenced by European and World Health Organization policy frameworks, this entity adopted the Policy for Improving the Health of the Population of the Republic of Srpska by 2020, which recognized the importance of addressing the social and environmental determinants of health and emphasized the need for intersectoral collaboration across government sectors [13]. Through this strategic document, health was framed as a cross-cutting policy objective, promoting intersectoral collaboration in line with HiAP principles on multisectoral governance and institutional capacity outlined in the Helsinki Statement [6,14]. Also, the principles of equity are emphasized in the Strategy of Social Inclusion of the Republic of Srpska for 2021–2027, which identifies children and young people, women, people living in poverty, people with disabilities, older people (commonly referred to in national strategic documents as persons in the third age of life), and members of national minorities as particularly vulnerable or marginalised. These groups are designated as such because structural barriers—in income, employment, access to health and social services, and social participation—place them at heightened risk of poorer health outcomes and social exclusion. To date, HiAP integration has not been systematically examined within the country’s scientific or academic community. Likewise, there is limited evidence of its structured application within governmental practice.

This study addresses that gap by assessing the extent to which HiAP principles are incorporated within sectoral legislation in the Republic of Srpska. The analysis covers legislation governing education, agriculture, emergency management, water management, urban and spatial planning, labour, environmental protection, transport, energy, and local self-government. Using an analytical framework focused on health-related provisions, cross-sectoral governance, social determinants and equity, evidence-informed policymaking, and monitoring mechanisms, this study seeks to contribute to understanding how legislation can support or constrain the implementation of HiAP within a complex governance system and to identify opportunities for strengthening HiAP implementation within complex governance systems.

## 2. Materials and Methods

### 2.1. Study Setting

The Republic of Srpska is one of the two constitutional entities of Bosnia and Herzegovina, alongside the Federation of Bosnia and Herzegovina, while the Brčko District functions as a self-governing administrative unit under the sovereignty of both entities [12]. According to the most recent demographic estimates, the Republic of Srpska has a population of approximately 1.1 million inhabitants [15]. Bosnia and Herzegovina have a highly decentralized governance structure, with limited competencies at the state level exercised through a tripartite Presidency and the Council of Ministers, while legislative authority is distributed across multiple institutional layers [16]. In contrast to the Federation of Bosnia and Herzegovina, which is further divided into cantons and municipalities, the Republic of Srpska operates as a more centralized entity, with legislative and executive powers vested in its National Assembly and Government, and administrative authority implemented at the municipal level [16].

### 2.2. Research Team

The research team consisted of 12 investigators with interdisciplinary expertise in medicine, epidemiology, public health, research methodology and environmental health. Drawing on this expertise, the team conducted a structured panel discussion to map the regulatory landscape, identifying key sectors and areas of governance that influence human health. To limit the subjectivity inherent in expert panels, the panel worked from a written protocol, all decisions on sector selection and coding were made by at least two members and documented, and coding was anchored to a priori operational definitions and a shared coding manual (Section 2.5). The team included members with formal training in law and public health policy who contributed to the interpretation of statutory provisions; nonetheless, the absence of an independent external legal reviewer is acknowledged as a limitation.

### 2.3. Regulation Prioritization and Analysis

At first, the team identified 29 key sectors including: water management, food production and trade, rural development, spatial and urban planning, tourism, construction, economy, transport, social welfare, family support and demographic policy, local governance, environment, justice and criminal law, human and minority rights, defense and security sector, labour and employment, veterans’ and social affairs, sport, culture and media, fire safety, hospitality, public sanitation, occupational safety and health, emergency preparedness, industrial development, religious and spiritual values, education, telecommunications, energy sector and agriculture.

After mapping of health-relevant sectors, the next step involved prioritizing regulations to identify ten key areas of focus. This prioritization was guided by several criteria, including the estimated public health significance, the team’s existing expertise, and prior experience. In some cases, related sectors previously identified were grouped together to streamline the analysis. Each area was then examined individually, and for more complex domains, specific sub-areas of interest were further delineated. Through this process, the following priority areas were highlighted: education; energy sector; agriculture (including food production and safety, as well as producer protection); water management (water safety); urban planning, spatial planning, and rural development; transport (road and rail safety); labour, occupational health and safety, including burnout and workplace harassment prevention; emergency preparedness; local governance; and environmental protection (general, air, waste, and soil). Prioritisation was intended to define a feasible and coherent analytical scope rather than to rank sectors by their absolute importance for health, and the criteria—estimated public health significance, the breadth of the determinants of health a sector regulates, and the analytical tractability of its statutory framework—were agreed and recorded before sector-level coding began. The selected sectors correspond to those most commonly identified in the HiAP literature as carrying substantial health determinants (transport, environment, education, labour, water, food, emergency management, energy, spatial planning and local governance). Several health-relevant sectors that were not retained as standalone domains—notably social welfare, justice, human and minority rights, housing-related construction, culture and media, and telecommunications—were either subsumed under a retained domain (for example, social protection within labour and local governance, and construction within spatial planning) or set aside to keep the first iteration of the instrument manageable. Their exclusion does not imply that they are unimportant for the social determinants of health, and their inclusion is identified as a priority for future iterations of this work. Because the priority set was shaped in part by the team’s expertise, the possibility of selection bias cannot be excluded and is acknowledged among the study limitations.

### 2.4. Analytical Tools and Instruments

Given the large number of regulations in certain sectors, the analysis was limited to laws, while sub-legal acts, strategies, and similar documents were excluded. A dedicated regulatory analysis instrument was developed to support the study. The instrument captured general information about each regulation, including the country or entity, date of adoption, adopting authority, regulation title and structure, subject matter, and identification of specific sections according to the analytical framework. Following the development of the initial draft, the instrument was analysed and optimized within the main research team and subsequently reviewed by all team members. The final version was pre-tested on a selected regulation and subsequently completed. Application of the instrument involved entering data for each regulation and extracting relevant sections according to the components of the analytical framework, enabling a systematic and comparable assessment across priority sectors. The unit of analysis was the primary law (zakon). Inclusion criteria were: (i) a legal act with the force of primary legislation that was in force in the Republic of Srpska at the time of analysis; (ii) adopted by the National Assembly of the Republic of Srpska; and (iii) falling within one of the ten prioritised sectors. Exclusion criteria were: (i) sub-legal acts (bylaws, rulebooks, ordinances), strategies, action plans and other policy documents; (ii) legal acts no longer in force; and (iii) laws outside the prioritised sectors. Where a law had been amended, the consolidated version in force at the time of analysis was used. Pre-testing on a single law led to minor refinements of the instrument—principally the addition of worked examples distinguishing explicit from implicit coding and clarification of the fields capturing institutional and monitoring arrangements—while no analytical category was added or removed. The full instrument and coding manual are available from the corresponding author on request, and the complete coded matrix is provided as Supplementary Table S1.

### 2.5. Analytical Framework

Based on the World Health Organization’s 2014 Health in All Policies (HiAP) Framework for Country Action, an analytical framework was developed to guide the assessment of regulations. The framework includes the following components:

1. Consideration of health outcome—the extent to which a regulation directly or indirectly considers health, well-being, prevention of disease and injury, or broader population-level health outcomes.
2. Intersectoral governance—institutional mechanisms that allocate joint responsibility for the formulation, implementation, and supervision of policies across different sectors.
3. Health equity and social determinants of health—identification of health outcome disparities across population and the adoption of strategies addressing the needs of vulnerable or high-risk populations.
4. Use of health evidence and assessment—integration of health information, epidemiological evidence, surveillance mechanisms and health impact assessments in policy design or implementation.
5. Monitoring, evaluation, and accountability—mechanisms for monitoring, documenting, or evaluating health outcomes as well as for assigning accountability for the health impacts of policies.

In addition to assessing compliance with these criteria, the review also identified examples of good practice and opportunities for regulatory improvement. Each component was scored for every regulation as implicitly included, explicitly included, or not included, providing a systematic basis for comparison across sectors and domains.

Operational definitions of the three coding categories were established a priori and applied uniformly across all legislative acts. A component was coded as “explicitly included” when the legal text contained an unambiguous, directly stated provision that named the relevant HiAP element using health-specific terminology (e.g., “protection of human health,” “health impact,” “vulnerable groups,” “intersectoral cooperation with the Ministry of Health”) and that articulated a corresponding obligation, mechanism, indicator, or institutional arrangement. A component was coded as “implicitly included” when the legal text addressed the relevant HiAP element only indirectly—through proxy concepts such as “safety,” “quality of life,” “environmental protection,” “general welfare,” “social protection,” or “cooperation with competent authorities”—without naming health, identifying a specific health outcome, designating vulnerable groups, or stipulating health-related monitoring obligations. In other words, the implicit code captured situations in which a plausible health-relevant interpretation could be supported by the wording, but the legislator did not articulate the health dimension as such. The “not included” code was reserved for laws in which neither explicit nor proxy formulations could be identified for the component in question.

To support coding reliability, each law was independently reviewed by two members of the research team using the structured analytical instrument. Coders worked from the same coding manual, which contained the operational definitions above together with worked examples for each of the five components. After independent coding, the two reviewers compared their assessments component by component. Initial agreement was high across the 110 component-level decisions (22 laws × 5 components), with full concordance reached on the great majority of items at the first round; the principal source of initial disagreement involved the boundary between “explicit” and “implicit” coding for laws that referred to “safety” or “environmental protection” without naming health. Discrepancies were resolved through structured discussion against the operational definitions, and any item that could not be reconciled by the two coders was referred to a third reviewer for adjudication; the consensus code was then entered into the analytical matrix. The full coded matrix is presented in Supplementary Table S1. Reliability was assessed through the consensus procedure described above rather than through a chance-corrected coefficient; a formal inter-rater statistic (for example, Cohen’s kappa) was not pre-specified and is therefore not reported, which we note as a methodological limitation. The disagreements that did arise were concentrated almost entirely on the explicit–implicit boundary rather than on the presence or absence of a component, indicating that the principal source of measurement uncertainty was the gradation of how clearly health was named, not whether a relevant provision existed. Future applications of the instrument should pre-register a chance-corrected reliability metric with reporting of component-specific agreement.

### 2.6. Results Analysis

The analysis encompassed the quantification and structural profiling of the included legislative acts, alongside with assessment of their alignment with the five predefined framework criteria. Particular attention was given to identifying examples of good practice with potential for cross-sectoral transfer. In addition, the study explored opportunities to enhance both the legislative framework and the applied analytical approach, including further refinement of the research instrument to ensure more comprehensive integration of all five components.

Variability across and within sectors was assessed by tabulating the coded matrix in two complementary ways. For between-sector variability, frequencies of “explicit,” “implicit,” and “not included” codes were aggregated by sector for each of the five HiAP components, allowing the proportion of explicit integration to be compared across the ten priority sectors. For within-sector variability, the coded profiles of laws belonging to the same sector were compared row by row, and a sector was considered internally heterogeneous when laws within it received divergent codes (for example, “explicit” for one law and “not included” for another) on the same component. To complement the descriptive distribution, an exploratory law-level summary score was derived for each act by counting the number of components (out of five) coded as “explicitly included”; this score was used solely as a heuristic for ranking laws on a continuum from comprehensive to minimal HiAP integration, not as a statistical test.

Building on this summary score, two qualitative categories were identified to inform the discussion of opportunities and gaps. Laws were flagged as “exemplary” (or “unusually hopeful”) when at least four of the five HiAP components were coded as “explicitly included” and the remaining component was coded as “implicitly included,” signalling broad and operationally specific integration of HiAP principles. Laws were flagged as “problematic” when no component, or only one component, was coded as “explicitly included,” or when three or more components were coded as “not included,” signalling that the legal text neither names health outcomes nor establishes the supporting governance, equity, evidence, or accountability mechanisms expected under the HiAP framework. These two categories did not generate additional quantitative analyses; rather, they were used to anchor the qualitative interpretation of sector-level patterns, to select the cases discussed under “Exemplary Practices in HiAP Implementation” (Section 3.8) and “Opportunities for Strengthening HiAP Integration” (Section 3.9), and to ensure that the legislative recommendations were grounded in concrete textual evidence rather than in general impression.

## 3. Results

### 3.1. Overview of the Analyzed Regulatory Framework

A mapping of legal instruments was carried out across the priority sectors identified in the Republic of Srpska. Although the original plan included laws, bylaws, regulations, strategies, and plans, the mapping exercise revealed that framework laws and subordinate legal acts constituted the vast majority of relevant instruments. To maintain consistency and comparability, the analysis was therefore confined to laws. In total, twenty-two sectoral laws were examined, covering energy, agriculture, labour, transport, urban planning, emergency management, water management, environmental protection, education and local governance (Table 1). Most of these laws were adopted between 2006 and 2017. The earliest in the sample is the Law on Agricultural Land and Law on Waters from 2006, while the most recent—the Law on Preschool Education—date from 2025. This distribution reflects a period of considerable legislative activity, with many of the analysed laws having undergone revisions in recent years.

In the energy sector, four laws were adopted between 2009 and 2022. The Law on Energy was last amended in 2023, and the Law on Geological Explorations in 2024. Agricultural legislation consists of three laws adopted between 2006 and 2017, with the Law on Agriculture revised most recently in 2025. The Law on Labour, originally adopted in 2015, was updated in 2023. Transport legislation shows a consistent pattern: the Law on Road Traffic Safety (2011, revised 2021), the Law on Road Transport of Passengers and Goods (2017, revised 2023), and the Law on Railways (2017, revised 2022). The Law on Spatial Planning and Construction, adopted in 2013, was revised in 2019, while the Law on Protection and Rescue in Emergency Situations, from 2012, was amended in 2017. The Law on Waters, dating from 2006, was last revised in 2017.

Environmental legislation includes the framework Law on Environmental Protection (2012, revised 2020), alongside the Law on Waste Management (2013, revised 2021) and the Law on Air Protection (2011, revised 2017). The education sector is represented by four laws adopted between 2018 and 2025, the most recent being the Law on Preschool Education from 2025. The Law on Local Self-Government, adopted in 2016, was revised in 2025. Taken together, these data point to sustained legislative activity across multiple policy domains, with framework laws serving to establish regulatory structures, define institutional responsibilities, and set out general objectives. Nevertheless, explicit references to health or population well-being are distributed unevenly across sectors.

### 3.2. Integration of HiAP Framework Components

**Figure 1:**
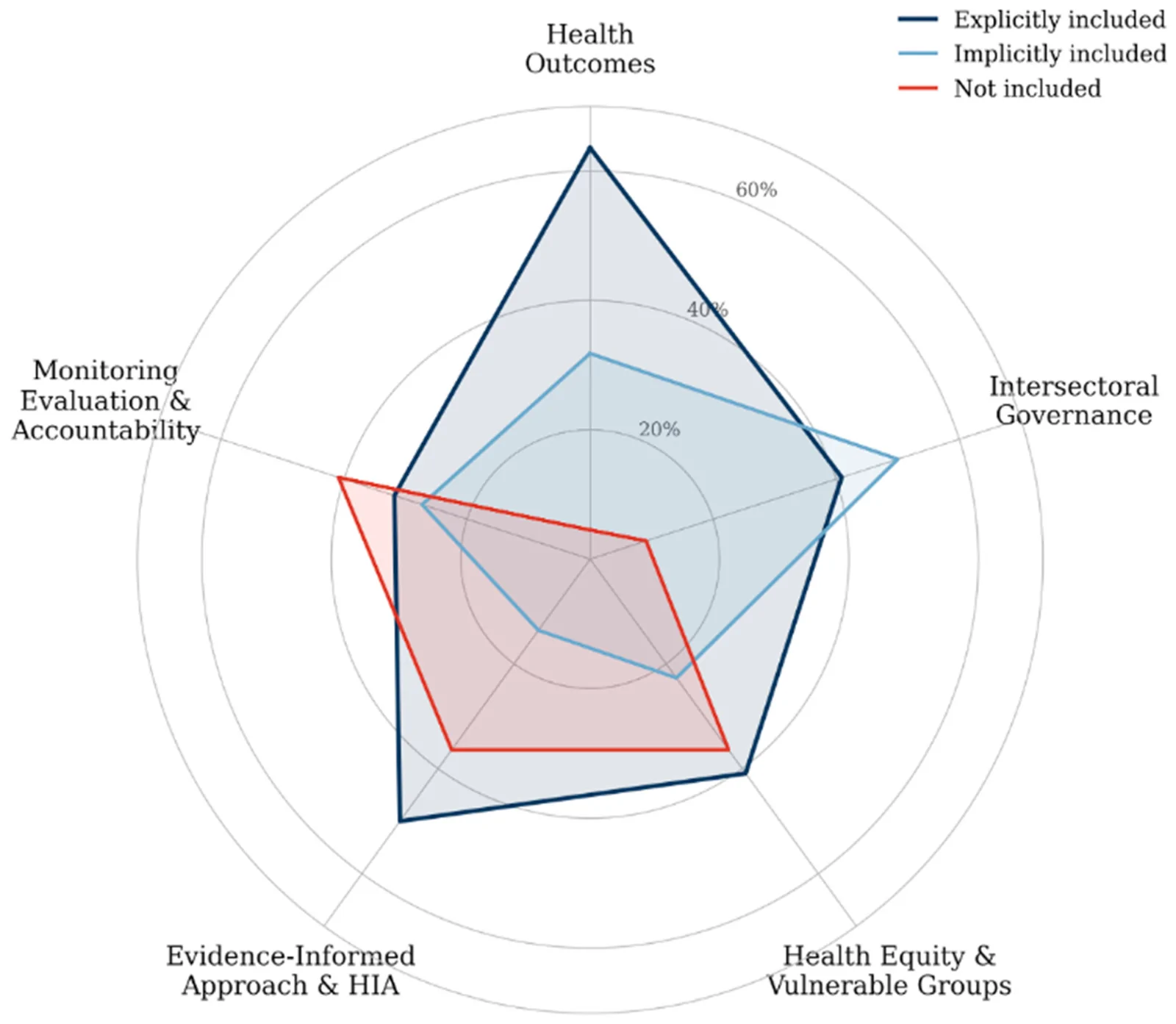
Distribution of HiAP component integration across the 22 analysed laws (percentage of laws coded as explicitly included, implicitly included, and not included for each of the five framework components). Values correspond to Table 2.

Each of the 22 laws was assessed against five components drawn from the World Health Organization’s 2014 Health in All Policies Framework for Country Action. The assessment used a three-point scale: “explicitly included” (direct reference with specific provisions), “implicitly included” (indirect consideration through related concepts without explicit articulation), or “not included” (no discernible reference). The aggregated results are presented in Table 2 and visualised in Figure 1.

A more detailed breakdown of how each individual law performs against the five HiAP components is provided in Supplementary Table S1. This disaggregated view reveals the considerable variation that exists not only across sectors but also among laws within the same sector (Supplementary Table S1).

Between-sector variability was inferred from Supplementary Table S1 by tabulating, for each sector, the proportion of component-level codes (out of all 5 components × the number of laws in the sector) that were marked as “explicitly included.” The transport sector showed the highest density of explicit codes, with 13 of 15 component decisions across its three laws (the Law on Road Traffic Safety, the Law on Road Transport of Passengers and Goods, and the Law on Railways) classified as explicit. The environment sector also performed strongly, with 9 of 15 component decisions explicit across the Law on Air Protection, the Law on Environmental Protection, and the Law on Waste Management. By contrast, the energy sector showed the highest concentration of implicit and absent codes (only 5 of 20 component decisions explicit, distributed unevenly across four laws), and the urban planning and water management sectors—each represented by a single framework law—contained no explicit codes at all on any of the five components. This sector-level distribution is what the manuscript refers to as between-sector variability: the same five HiAP components yield substantially different code profiles depending on the policy domain in which they are evaluated.

Within-sector variability was identified by reading Supplementary Table S1 row by row within each multi-law sector and locating components on which laws of the same sector received divergent codes. The starkest example is the education sector, where the Law on Preschool Education was coded as “explicitly included” on four of the five components, while the Law on Higher Education was coded as “not included” on all five. Similar within-sector heterogeneity was found in agriculture, where the Law on Food was coded as “explicitly included” on all five components, the Law on Agricultural Land showed a mixed profile, and the Law on Agriculture received no explicit codes at all. The energy sector also displayed internal contrast: the Law on Geological Explorations and the Law on Mining contained explicit codes on health outcomes and on the use of health evidence, whereas the Law on Energy and the Law on Renewable Energy Sources did not contain any explicit codes on these components. This pattern—sectors that are internally inconsistent rather than uniformly weak or uniformly strong—is what the manuscript refers to as within-sector variability, and it is the principal reason why aggregate sector labels alone are insufficient to characterize HiAP integration.

### 3.3. Consideration of Health Outcomes

Explicit references to health outcomes appear in 14 laws (63.6%), most commonly in transport, labour, education, and environmental protection legislation. In energy, agriculture, water management, and urban planning, health tends to be addressed indirectly, framed within concepts of safety, environmental protection, or quality of life.

The finding that laws in some sectors address health only indirectly is a direct expression of the “implicitly included” coding category, applied to the “Consideration of Health Outcomes” component. As specified in the operational definitions in Section 2.5, this code was assigned when a law neither named health nor stipulated a health-related obligation, yet contained provisions whose object of protection plausibly corresponds to a determinant of health (e.g., “safety,” “quality of life,” “environmental protection,” or “sustainable development”). The laws that received an “implicit” code on this component are precisely the laws referred to as addressing health “indirectly” in the preceding paragraph. Concretely, this corresponds in Supplementary Table S1 to the Law on Energy, the Law on Renewable Energy Sources, the Law on Agriculture, the Law on Agricultural Land, the Law on Spatial Planning and Construction, the Law on Waters, and the Law on Local Self-Government, all of which were coded as “implicitly included” for health outcomes; the Law on Higher Education, by contrast, was coded as “not included” because no provision could be reasonably construed as relating to health, even by proxy. Reframing the “indirect” finding in these terms makes clear that “addressed indirectly” is not a softer description of the same legislative content as “explicitly included,” but a substantively different category in which the health dimension must be inferred by the reader rather than read off the legal text. From a HiAP standpoint, the operational consequence is that such provisions are unlikely to generate enforceable health obligations, dedicated indicators, or accountable responsibilities, and therefore tend to translate into weaker downstream coding on the four remaining components, as documented in Section 3.4, Section 3.5, Section 3.6 and Section 3.7.

The Law on Labour explicitly guarantees protection of life and health at work. Transport legislation offers strong integration: the Law on Road Traffic Safety aims to reduce injuries and fatalities, while the Law on Railways establishes safety systems addressing serious injuries. In energy, health considerations remain largely implicit. The Law on Energy touches on health through environmental protection and sustainable development objectives, but does not define health indicators. Agricultural legislation shows mixed patterns: the Law on Food explicitly aims to protect human life and health, while the Law on Agriculture mentions health mainly through compliance with public health standards for subsidy eligibility. Environmental legislation addresses health in protective terms, with the Law on Air Protection basing air quality limits on health criteria and mandating protective measures during pollution episodes. In education, the Law on Preschool Education comprehensively addresses children’s physical and mental health and mandates health professionals in preschools, while the Law on Higher Education contains no health references. The Law on Protection and Rescue in Emergency Situations explicitly prioritises human life through medical assistance and evacuation of vulnerable populations.

### 3.4. Intersectoral Governance Mechanisms

Provisions for intersectoral governance appear in 20 laws (90.9%), with explicit mechanisms in 9 laws (40.9%). Formalised governance is most evident in transport, emergency management, and food safety legislation.

The Law on Road Traffic Safety establishes a multi ministerial Council for Traffic Safety (transport, interior, health, education) and a dedicated Agency. The Law on Protection and Rescue in Emergency Situations creates a multi sectoral system involving the Ministry of Interior, Civil Protection Administration, local governments, and citizens. The Law on Food formalizes collaboration through a Food Council with mandated representation from public health, veterinary, and agricultural institutions. The Law on Preschool Education mandates cooperation between preschools, health facilities, and social welfare centres. Implicit governance appears as general references to “cooperation” without specified mechanisms. The Law on Geological Explorations mentions cooperation with environmental and mining authorities but establishes no formal coordination. The Law on Energy and Law on Renewable Energy Sources assign responsibilities to various state bodies but lack formal cross sectoral arrangements. The Law on Higher Education contains no intersectoral governance provisions related to health.

### 3.5. Health Equity and Vulnerable Groups

This component shows the greatest variation. Explicit provisions appear in 9 laws (40.9%), while 5 laws (22.7%) address these issues implicitly, and 8 laws (36.4%) contain no such provisions.

The education sector demonstrates the most systematic incorporation of equity. The Law on Primary Education defines multiple vulnerable categories—children with developmental difficulties, learning disabilities, behavioural problems, and those disadvantaged by social, economic, or cultural factors—and mandates individualized programs and teaching assistants. The Law on Preschool Education establishes priority enrolment for children from vulnerable categories including social welfare recipients, children with rare diseases, children of disabled parents, single parent families, and minority communities. The Law on Labour provides detailed protections for pregnant women, new mothers, minors, persons with disabilities, and single parents. The Law on Air Protection explicitly identifies children, elderly persons, and chronic patients for special protective measures during pollution episodes. The Law on Protection and Rescue in Emergency Situations prioritizes these groups in evacuation planning. Implicit attention appears as general population protection without specific targeting. The Law on Waters and Law on Agricultural Land protect public health through quality controls but do not identify groups with heightened vulnerability. Notable absences of equity provisions were found in the Law on Geological Explorations, Law on Renewable Energy Sources, and Law on Agriculture.

### 3.6. Evidence-Informed Approach and Health Assessments

Evidence-based approaches appear in 14 laws (63.6%), with explicit requirements in 11 laws (50.0%), particularly in technical and risk-prone sectors.

The Law on Food mandates risk assessment, monitoring, and laboratory analysis using internationally recognized methods. The Law on Mining mandates workplace risk assessments and expert project review. The Law on Road Traffic Safety requires data collection from multiple sources with sex disaggregated injury data, and strategy development based on safety analysis. The Law on Environmental Protection requires health considerations in environmental impact assessments. The Law on Air Protection bases limit values on health criteria with systematic monitoring. The Law on Protection and Rescue in Emergency Situations requires vulnerability assessments for emergency planning. Implicit reliance appears as planning requirements without explicit health evidence mandates. The Law on Preschool Education uses developmental monitoring but does not call for epidemiological data; the Law on Waters requires sanitary studies but not systematic health impact assessment. No law explicitly mandates Health Impact Assessment as a distinct tool.

### 3.7. Monitoring, Evaluation, and Accountability for Health Outcomes

This component represents the most significant gap. Explicit mechanisms appear in 7 laws (31.8%), implicit monitoring in 6 laws (27.3%), while 9 laws (40.9%) contain no relevant provisions.

The Law on Road Traffic Safety mandates annual reporting with injury data for policy evaluation. The Law on Air Protection establishes continuous monitoring with public data dissemination and accountability for pollution exceedances. The Law on Preschool Education requires health professionals to monitor children’s development and carry out annual employee examinations, with director accountability. The Law on Waters mandates systematic drinking water testing with corrective measures. The Law on Food establishes sampling, rapid alert systems, and crisis procedures. Implicit monitoring focuses on compliance rather than health outcomes. The Law on Mining includes inspection oversight but no systematic tracking of miners’ health. The Law on Energy requires administrative supervision without explicit health indicators. An absence of provisions was notable in the Law on Higher Education, Law on Agriculture, and Law on Agricultural Land.

### 3.8. Exemplary Practices in HiAP Implementation

Several legislative provisions represent exemplary practice. The Law on Road Traffic Safety establishes a multi ministerial Council for Traffic Safety, a dedicated Agency, and mandated coordination with local government and research bodies, creating permanent structures for sustained intersectoral collaboration. In education, the Law on Preschool Education and Law on Primary Education identify diverse vulnerable categories with affirmative measures including priority enrolment, individualized programs, and teaching assistants, while integrating health professionals within educational settings. The Law on Food adopts risk analysis methodology requiring independent, transparent assessment with internationally recognized methods, while the Food Council’s mandatory public health representation institutionalizes health expertise in food policy governance. The Law on Air Protection bases air quality limits on health criteria, with special protective measures for children, elderly, and chronic patients during pollution episodes, and systematic monitoring with public disclosure. The Law on Labor provides twelve-month maternity leave, half time work rights for parents of children with severe developmental difficulties, prohibitions on hazardous work for vulnerable groups, and regular health examinations. The Law on Protection and Rescue in Emergency Situations prioritizes human life and health through medical assistance, evacuation procedures prioritizing vulnerable groups, and explicit intersectoral coordination requirements.

### 3.9. Opportunities for Strengthening HiAP Integration

The analysis reveals several opportunities for enhancing HiAP integration. Laws in energy, agriculture, and water management could be amended to explicitly identify health protection as a primary policy objective rather than an incidental co benefit. The Law on Energy could recognize the health benefits of renewable energy transition, including reduced air pollution and associated respiratory diseases. The Law on Secondary Education could incorporate mental health promotion and psychosocial support for adolescents. Laws could also move from general references to “health protection” to specifying measurable health outcomes, such as waterborne disease incidence targets in the Law on Waters or nutrition related indicators in the Law on Agriculture.

Intersectoral governance can be strengthened by moving from implicit “cooperation” to formal, legally mandated bodies with defined composition and accountability, adapting the Council for Traffic Safety model to sectors like urban planning and food policy. Sectoral laws should mandate health authority consultation in policy development and major project approvals.

Regarding health equity, laws should explicitly identify vulnerable populations requiring special protection—such as children, pregnant women, and immunocompromised individuals in water regulations, or agricultural workers in pesticide regulations. Building on the education sector model, affirmative measures including priority access and equity focused local health plans should be incorporated.

HIA should be explicitly mandated as a distinct tool for prospective evaluation of policies, plans, and projects. Sectoral planning processes should systematically incorporate epidemiological data and public health research findings. The most critical gap—monitoring and accountability—requires amending sectoral laws to mandate systematic tracking of health outcomes relevant to each sector, such as longitudinal health surveillance of miners or pesticide related health effects in agricultural populations. Sectoral annual reports should include population health indicators, as modelled by the Law on Air Protection. Clear accountability mechanisms with designated responsible authorities should be established, drawing on the Law on Labour model for occupational health violations (Table 3).

Framework HiAP legislation or strategy should be considered to establish principles, mechanisms, and accountability structures across all sectors. Capacity building provisions must include training for non-health sector professionals in health determinants and HiAP approaches. Stakeholder participation should be strengthened through meaningful involvement of civil society, health advocacy organizations, and community representatives. Legislative reforms must align with international commitments including the Sustainable Development Goals, WHO European Region HiAP initiatives, and relevant European Union directives. In relation to the 2030 Agenda, the HiAP elements examined here map most directly onto Sustainable Development Goal (SDG) 3 (Good Health and Well-being), and in particular target 3.9 on reducing deaths and illness from hazardous chemicals and air, water and soil pollution, target 3.6 on road traffic injuries, and target 3.d on early warning and risk management; onto SDG 17 (target 17.14 on enhancing policy coherence for sustainable development), which corresponds to the intersectoral-governance component; and onto the equity-oriented commitments of SDG 10 (target 10.2 on social, economic and political inclusion) and SDG 5, which align with the health-equity and vulnerable-groups component. The cross-sectoral scope of the analysis also intersects with the health determinants addressed by SDGs 2, 4, 6, 8 and 11. Framing legislative reform against these specific targets would allow progress on HiAP integration to be reported within existing national SDG monitoring rather than as a separate exercise.

## 4. Discussion

### 4.1. Overall Interpretation of Findings

This study provides the first systematic assessment of HiAP integration within sectoral legislation in Republic of Srpska, identifying legal entry points for strengthening health equity, health impact assessment, and accountability mechanisms. It contributes to the limited evidence on HiAP implementation in decentralized governance settings and offers an analytical approach potentially transferable to other contexts. Methodologically, the present study belongs to the established public health tradition of analysing the content of law as data. This tradition—variously described as policy surveillance and legal epidemiology—treats the systematic coding of statutory text against defined indicators as a legitimate object of study that precedes, and is distinct from, the evaluation of enforcement or health outcomes [10,11]. Earlier applications have largely been single-issue and quantitative, scoring the strength of laws within one domain such as tobacco control, clean indoor air, or school nutrition [11], or have examined how health is framed within the legislation of a single sector, as in the qualitative content analysis of land-use planning law in New South Wales [17]. The present study differs from these precedents in two respects: it applies a common HiAP-derived framework simultaneously across ten sectors rather than within one, and it situates the analysis in a decentralised, post-conflict, transitional governance setting that is under-represented in this literature. Our results are consistent with the cross-sector finding of that literature—that health is most explicitly codified in domains with long-standing statutory health links (transport, labour, education and environmental protection) and only implicitly in domains traditionally regarded as outside the health remit (energy, agriculture and water management) [10,17]—while extending it to a multi-sector legislative corpus that had not previously been examined in this way. Consistent with the premises of this approach, the analysis characterises what the law says and does not, by design, measure implementation, enforcement, or downstream health outcomes; these are treated as the necessary next stages of inquiry rather than as questions the present design can answer.

The findings indicate that health considerations are broadly recognized across sectors, confirming that health is not viewed exclusively as a responsibility of the health sector, but as a cross-cutting societal value. However, the depth, consistency, and operationalization of HiAP components vary substantially across laws and sectors, revealing a pattern of partial and uneven integration rather than a fully institutionalized HiAP approach. The strongest performance was observed in the explicit consideration of health outcomes and intersectoral governance mechanisms, while monitoring, evaluation, and accountability for health outcomes emerged as the most significant systemic gap. This pattern suggests that although health is increasingly acknowledged at the normative and declarative level, it is less consistently translated into measurable obligations, enforceable responsibilities, and long-term policy learning mechanisms. This trend aligns with international literature highlighting that HiAP often remains partially implemented—with clear intersectoral collaboration, but without permanently institutionalized solutions to ensure a sustainable impact on health outcomes [18]. Furthermore, the incorporation of health considerations across sectors is context-dependent and often shaped by longstanding administrative priorities [19]. Although many local programs have succeeded in establishing formal collaboration and inclusive processes, most of them still do not achieve full institutionalization of health-oriented policies [20].

### 4.2. Health Outcomes and Cross-Sectoral Determinants Within HiAP Frameworks

Clear sectoral differences were identified, reflecting both the inherent relationship between specific policy domains and health, as well as the historical development of regulatory frameworks. Our findings align with international observations reported by WHO, showing that HiAP integration is strongest in sectors with well-established health links—such as transport, education, environmental protection, emergency management or labour—where health objectives are often explicitly embedded in policy and governance structures [21,22]. In contrast, sectors such as energy, agriculture and water management or higher education generally address health only indirectly or as a secondary concern [13,21,22]. Such indirect framing limits the visibility of health implications in policy implementation and reduces accountability for health outcomes, despite the substantial influence these sectors exert on population health through air quality, occupational exposure, nutrition and other social determinants [21,22]. Within the analysed legislation, the education sector stands out as a particularly illustrative example. More recent education laws incorporate provisions related to health equity, child development and cooperation with health and social services in a structured and comprehensive manner. This temporal pattern suggests that newer legislative frameworks are more likely to reflect contemporary policy paradigms emphasizing social determinants of health and whole-of-government approaches.

Evidence from international HiAP initiatives demonstrates that health considerations can be effectively translated into measurable improvements in health outcomes when supported by strong institutional frameworks [23]. One of the most frequently cited examples is the HiAP initiative implemented in South Australia, where the government introduced the Health Lens Analysis (HLA) methodology to systematically assess how policies in areas such as urban planning, transport and environmental regulation affect health determinants [24,25]. Evaluations of this initiative indicate that integrating health considerations into diverse policy areas contributed to improved policy coherence and measurable progress in determinants of health, including active transport, healthier urban environments and improved access to services [24,25]. The success of this approach has been widely attributed to strong political commitment, centralized coordination within government and the institutionalization of health considerations within routine policy development processes [23,24,25].

Intersectoral collaboration is widely recognized as a cornerstone of effective HiAP implementation [23]. Countries such as Finland have long institutionalized mechanisms for cross-sectoral cooperation through national public health strategies addressing the social determinants of health [23,26]. Finnish governance structures promote collaboration between health authorities and sectors such as education, labour, transport and social welfare, supported by formal coordination bodies and shared policy objectives [23,26]. Similarly, the establishment of the HiAP Task Force in California brought together representatives from multiple state agencies to integrate health perspectives into policies related to housing, transportation, food systems and environmental protection [9,27]. These experiences suggest that sustained intersectoral collaboration is most effective when supported by formal governance mechanisms, clear institutional mandates and policy frameworks that align health objectives with broader social and economic priorities [9,27].

### 4.3. Health Equity as an Inconsistently Addressed Dimension

Health equity emerged as the most unevenly integrated component of the HiAP approach across the analysed legislation. While certain laws—particularly in the sectors of education, labour, air protection and emergency management—explicitly recognize vulnerable population groups and introduce affirmative measures, a substantial number of laws address equity only implicitly or not at all. This inconsistency suggests that equity considerations are not yet systematically embedded as a standard legislative requirement. Similar inconsistencies have been documented in comparative policy analyses, demonstrating that, although health equity is increasingly acknowledged in policymaking, its systematic integration across sectors remains uneven and highly context-dependent [24]. As emphasized in the literature on the social determinants of health, policies outside the health sector play a decisive role in shaping population health inequalities, making cross-sector coordination essential for their effective reduction [28]. However, several studies have also highlighted persistent challenges in translating HiAP principles into measurable equity outcomes, as many initiatives continue to struggle with addressing health inequalities in practice [29].

The strong equity provisions observed in education legislation illustrate that the existing legal framework is capable of incorporating detailed recognition of vulnerable groups together with concrete support measures, such as priority access, adapted services and institutional assistance. However, the absence of similar provisions in sectors such as agriculture, energy and mining are particularly notable given the documented differential health impacts of these sectors on rural populations, low-income households and occupationally exposed workers. This gap underscores the importance of integrating an explicit distributional perspective in sectoral policymaking.

Several countries have successfully embedded equity considerations into national health strategies by explicitly linking health policies with broader social and economic policies. In Finland, for example, long-standing policy initiatives have focused on reducing socioeconomic health disparities into national public health strategies through coordinated policies across sectors such as employment, education, regional development and social protection [26,30,31]. Similarly, national policy frameworks in Norway emphasize structural interventions targeting the social determinants of health as a central mechanism for reducing health inequalities [14,32]. These approaches demonstrate that tackling health disparities requires coordinated action across multiple policy domains, supported by strong political commitment and long-term strategic planning [14,32].

### 4.4. Evidence Use Without Systematic Health Impact Assessment

Health Impact Assessment (HIA) is well established instrument for operationalizing the HiAP approach, as it provides a structured framework for systematically assessing the potential health consequences of policies across sectors [33]. In particular, HIA is focused on assessing how policy decisions may differentially affect population groups and contribute to health inequalities [33]. Nevertheless, despite its conceptual importance, the global HIA implementation remains uneven and highly dependent on supportive institutional frameworks and political commitment [34]. In many contexts, including those examined in this study, health considerations are frequently embedded within environmental or technical assessments rather than addressed through dedicated HIA processes [35]. While such assessments may capture certain environmental or safety risks, they often provide only a partial understanding of broader social determinants of health and their distribution across population groups [35].

Although the analysed legislation demonstrates a considerable reliance on scientific evidence, data collection and technical assessments—particularly in sectors addressing environmental risks, food safety, transport safety and emergency preparedness—this reliance remains fragmented and largely confined within sector-specific regulatory frameworks. None of the analysed laws explicitly mandate HIA as a standardized policy instrument. Instead, health issues are typically considered within environmental impact assessments, risk analyses or other technical evaluations. These approaches may insufficiently capture wider social determinants and the distribution of health impacts across population groups. The absence of a formal HIA requirement therefore represents a missed opportunity to harmonize the use of evidence across sectors and to ensure that potential health effects are systematically considered during policy development rather than addressed retrospectively or incidentally [10]. Consequently, HIA remains an underutilized tool in public health practice, often applied only in individual projects or ad hoc initiatives rather than systematic institutional procedures [10].

The importance of evidence-informed policymaking has been widely emphasized in the HiAP literature. Several jurisdictions demonstrate how institutionalized frameworks can strengthen the integration of scientific evidence into policymaking. In South Australia, for example, the HLA approach systematically evaluates potential health implications of policies before implementation, supporting coordinated cross-sector decision-making [24,25]. Similarly, public health policy development in Sweden strongly relies on national health indicators, surveillance data and epidemiological research to guide priorities related to social determinants of health [22,31]. These experiences suggest that structured mechanisms for integrating scientific evidence into policy processes are essential for ensuring that health considerations are meaningfully incorporated across sectors, transforming HiAP from a conceptual framework into actionable policy practice.

### 4.5. Monitoring, Accountability, and the Implementation Gap

The most critical weakness identified in this analysis relates to monitoring, evaluation and accountability for health outcomes. Although many laws establish inspection mechanisms, compliance controls or forms of administrative oversight, these arrangements tend to focus primarily on procedural or technical compliance rather than on assessing actual health effects at the population level. Only a limited number of laws require systematic collection and reporting of health-relevant indicators, and even fewer explicitly link such indicators to policy evaluation or corrective action. This implementation gap suggests that health objectives, once incorporated in legislation, may remain largely symbolic unless supported by clearly defined indicators, reporting obligations and institutional accountability mechanisms. The contrast between relatively robust monitoring frameworks in sectors such as air protection and road traffic safety and their near absence in areas such as agriculture and higher education further highlights the importance of legally mandated health indicators.

Similar discrepancies between procedural oversight and outcome-oriented evaluation have been reported in the broader HiAP and HIA literature. International frameworks emphasize that effective HiAP implementation requires systematic monitoring, evaluation, and reporting of health indicators across sectors [14]. However, empirical studies indicate that many policies rely primarily on compliance-oriented administrative oversight rather than on the evaluation of population health outcomes [11,18]. The HIA literature likewise underscores that although monitoring of health determinants and outcomes is considered a core element of impact assessment, such evaluations are rarely implemented due to limitations in data systems, insufficiently defined indicators and weak institutional accountability mechanisms [17].

Successful HiAP initiatives often include mechanisms for monitoring and evaluating the health impacts of policies over time. In several national contexts comprehensive frameworks for tracking health determinants and population health indicators across policy sectors have been developed. In South Australia, evaluation processes were incorporated into the HiAP initiative through systematic policy reviews and ongoing monitoring of selected health determinants [24,25]. Similarly, national public health strategies in Finland include long-term monitoring systems that track population health indicators and support periodic policy evaluation [26]. These experiences demonstrate that effective monitoring and evaluation require robust data systems, clearly defined indicators and institutional structures responsible for assessing policy outcomes [14,22]. Without such mechanisms, the integration of health considerations into legislation risks remaining largely symbolic rather than translating into measurable improvements in population health.

Beyond documenting that these gaps exist, it is worth considering why they recur in the pattern observed. Three explanations are plausible and mutually compatible. First, the gaps track the historical development of each sector’s statutory base: domains in which health protection has long been an explicit regulatory purpose (transport safety, occupational health, communicable-disease and environmental control) have accreted detailed health language, indicators and oversight bodies over successive revisions, whereas domains framed primarily around production, infrastructure or economic objectives (energy, agriculture, water abstraction) inherited a vocabulary of “safety,” “quality” and “sustainability” in which health remains implicit. The temporal signal in the data—more recent laws, such as the 2025 Law on Preschool Education, being among the most explicit—is consistent with this account. Second, the monitoring and accountability deficit reflects a generic feature of framework legislation rather than indifference to health: framework laws characteristically delegate operational detail, including indicators and reporting duties, to subordinate acts that were outside the scope of this analysis, so part of the apparent gap may reside in instruments not examined here. Third, the cross-sectoral mechanisms that HiAP requires—joint mandates, shared indicators, formal coordinating bodies—impose coordination costs that are difficult to legislate within a single sectoral statute and that, in a decentralised system, must be reconciled across ministries with distinct mandates and budgets. These explanations matter for feasibility. The international exemplars discussed above (South Australia, Finland, California, Sweden, Norway) achieved institutionalisation under conditions—sustained political commitment, centralised whole-of-government coordination, mature data infrastructure and fiscal headroom—that differ markedly from the administrative, fiscal and political realities of the Republic of Srpska. Their experience is therefore better read as evidence that specific mechanisms (a standing intersectoral council, a health-lens procedure, mandated indicators) can work, rather than as templates for direct transfer. This is precisely why the recommendations developed below are framed as incremental, statute-specific amendments anchored in provisions already present in Republic of Srpska law, rather than as a call for wholesale adoption of any single foreign model.

### 4.6. Strengths and Limitations

This study has several important strengths. To our knowledge, it represents the first systematic assessment of the integration of HiAP principles into sectoral legislation in the Republic of Srpska, addressing an important empirical gap. A major strength is the use of a structured analytical framework based on the WHO HiAP Framework for Country Action, enabling systematic assessment across multiple legislative domains. The interdisciplinary research team, broad cross-sectoral scope covering ten sectors and 22 laws, and the identification of good practices and regulatory gaps further strengthen the study. In addition, the methodological approach developed in this research may support comparative legislative analyses in other jurisdictions.

Several limitations should be acknowledged. First, the analysis was limited to laws and did not include bylaws, strategies, policy documents, or implementation practices, all of which may contribute importantly to the operationalization of HiAP. In addition, the analysed legislation varied considerably in its level of detail. While some laws contain comprehensive substantive provisions, others primarily establish broad principles and rely on subordinate legislation for implementation. This reflects differing legislative approaches, ranging from detailed statutory regulation to framework laws that delegate operational provisions to bylaws. Although such approaches may offer either greater deliberative legitimacy or greater flexibility, respectively, they also influence the extent to which health-related considerations are explicitly captured within primary legislation. Furthermore, the focus on a single jurisdiction and a selected group of priority sectors may limit the generalizability of findings to other governance settings. Finally, although a structured analytical instrument was applied, the classification of provisions as explicit, implicit, or absent inevitably involved a degree of interpretive judgment. Most fundamentally, the study measures only the textual presence and framing of HiAP elements in primary legislation; by design it cannot establish whether coded provisions are enforced, funded, monitored in practice, or associated with any change in intersectoral practice or population health. The presence of explicit statutory language is therefore an indicator of legal opportunity, not of implementation, institutional readiness, or effectiveness, and a law coded as “explicitly included” may nonetheless be poorly implemented while a law coded as “implicitly included” may in practice be supported by effective administrative arrangements. Relatedly, the analysis does not capture the legal hierarchy and interaction between primary laws and the subordinate acts that often carry the operative detail, so the monitoring and accountability gap in particular may be partly an artefact of confining the corpus to primary legislation. The coding scheme also treats each law as a single unit and weights the five components equally; it does not account for differences in legal force, scope, or length between a major framework law and a narrow one, nor for the likelihood that some components (for example, monitoring and accountability, or Health Impact Assessment) are more consequential for health than a general health reference. The exploratory law-level summary score and the “exemplary”/“problematic” categories were defined by the authors for interpretive purposes and have not been externally validated. Finally, the broad “implicit” category, which credits proxy terms such as “safety” or “environmental protection” as health-relevant, may overstate the degree of latent HiAP alignment; the strict operational definitions and the separation of “implicit” from “explicit” coding were intended to contain, but cannot fully eliminate, this risk of interpretive inflation.

## 5. Conclusions

This comprehensive legislative analysis demonstrates that HiAP principles are partially embedded across sectoral laws in the Republic of Srpska, indicating that, despite an existing legal foundation, their integration remains incomplete and uneven. Nonetheless, these findings describe the content of legislation rather than institutional readiness; what they document is a set of existing legal entry points—not a demonstrated capacity for implementation—on which a more coherent and institutionalized HiAP approach could in principle be built. Targeted amendments that strengthen weak components—particularly in health equity, Health Impact Assessment and health-focused monitoring—offer a plausible starting point, but legislative change alone is unlikely to be sufficient; realising HiAP will also require subordinate regulation, resourcing, intersectoral coordination capacity, and implementation and outcome evaluation that lie beyond the text of primary law and beyond the scope of this study. The analysis further highlights the dual role of legislation as both an enabler and a constraint: explicit provisions on intersectoral collaboration, evidence use, and accountability support sustainable implementation, whereas implicit or narrowly framed health considerations limit meaningful integration.

Importantly, the identified examples of good practice demonstrate that closer alignment with the WHO HiAP framework is feasible through incremental, context-sensitive policy improvements. Beyond its national relevance, this study contributes to the limited body of evidence from post-conflict, transitional governance settings and offers a replicable methodological approach for assessing legislative alignment with HiAP principles. Greater dissemination of such experiences is essential for other countries and jurisdictions, in order to support knowledge transfer, identify effective strategies, and develop approaches for overcoming the practical challenges encountered during implementation.

## Figures and Tables

**Table 1:** Overview of the analysed legislation by sector.

No.	Law	Sector	Type	Adopted	Last Revision
1	Law on Energy	Energy	Law	2009	2023
2	Law on Geological Explorations	Energy	Law	2022	2024
3	Law on Renewable Energy Sources	Energy	Law	2022	-
4	Law on Mining	Energy	Law	2018	-
5	Law on Food	Agriculture	Law	2017	-
6	Law on Agriculture	Agriculture	Law	2009	2025
7	Law on Agricultural Land	Agriculture	Law	2006	2022
8	Law on Labour	Labour	Law	2015	2023
9	Law on Road Traffic Safety	Transport	Law	2011	2021
10	Law on Road Transport of Passengers and Goods	Transport	Law	2017	2023
11	Law on Railways	Transport	Law	2017	2022
12	Law on Spatial Planning and Construction	Urban Planning	Law	2013	2019
13	Law on Protection and Rescue in Emergency Situations	Emergency Management	Law	2012	2017
14	Law on Waters	Water Management	Law	2006	2017
15	Law on Waste Management	Environment	Law	2013	2021
16	Law on Air Protection	Environment	Law	2011	2017
17	Law on Environmental Protection	Environment	Law	2012	2020
18	Law on Primary Education	Education	Law	2022	-
19	Law on Preschool Education	Education	Law	2025	-
20	Law on Secondary Education	Education	Law	2018	-
21	Law on Higher Education	Education	Law	2020	-
22	Law on Local Self-Government	Local Governance	Law	2016	2025

**Table 2:** Comprehensive assessment of HiAP component integration across all reviewed laws (*n* = 22).

HiAP Component	Explicitly Included n (%)	Implicitly Included n (%)	Not Included n (%)
1. Consideration of Health Outcomes	14 (63.6%)	7 (31.8%)	1 (4.6%)
2. Intersectoral Governance Mechanisms	9 (40.9%)	11 (50.0%)	2 (9.1%)
3. Health Equity and Vulnerable Groups	9 (40.9%)	5 (22.7%)	8 (36.4%)
4. Evidence-Informed Approach and Health Assessments	11 (50.0%)	3 (13.6%)	8 (36.4%)
5. Monitoring, Evaluation and Accountability for Health	7 (31.8%)	6 (27.3%)	9 (40.9%)

**Table 3:** Illustrative, statute-specific proposals for strengthening HiAP integration in priority sectors of Republic of Srpska legislation.

Sector (Representative Law)	HiAP Component(s) Addressed	Proposed Legislative Amendment
Energy (Law on Energy; Law on Renewable Energy Sources)	Health outcomes; evidence	Add an explicit objective recognising the health co-benefits of the energy transition (reduced air pollution and respiratory disease); require health criteria in the licensing of major energy installations.
Agriculture (Law on Agriculture; Law on Agricultural Land)	Health outcomes; equity; monitoring	Specify measurable nutrition- and food-safety-related health objectives; identify occupationally exposed agricultural workers and rural low-income households as groups requiring protection; mandate surveillance of pesticide-related health effects.
Water management (Law on Waters)	Equity; monitoring	Name vulnerable users (children, immunocompromised persons) for special protection; set waterborne-disease incidence indicators and link them to corrective action, adapting the reporting model of the Law on Air Protection.
Urban and spatial planning (Law on Spatial Planning and Construction)	Health outcomes; intersectoral governance; evidence	Introduce a mandatory health consultation in the approval of major plans and projects; adopt a Health Impact Assessment/health-lens procedure for spatial plans.
Education (Law on Secondary Education; Law on Higher Education)	Health outcomes; equity	Incorporate mental-health promotion and psychosocial support for adolescents; extend explicit recognition of vulnerable students and student health provisions to higher education.
All sectors (framework provision)	Intersectoral governance; HIA; monitoring; accountability	Establish, by framework HiAP legislation or by amendment, a standing intersectoral coordinating body with defined composition and accountability (adapting the Council for Traffic Safety model); mandate Health Impact Assessment as a distinct prospective tool; require sectoral annual reports to include population-health indicators with designated responsible authorities.

## Data Availability

The data used in this study consist of publicly available legislative documents from the Republic of Srpska and the Autonomous Province of Vojvodina (Republic of Serbia). All analyzed laws are accessible through official government websites.

## Supplementary Materials

The following supporting information can be downloaded at: https://www.mdpi.com/article/10.3390/healthcare14142139/s1, Table S1. Assessment of Republic of Srpska Legislation Using the Health in All Policies Analytical Framework.
