# Correction: Mijović et al. Health in All Policies in Sectoral Legislation: A Content Analysis of Selected Laws in the Republic of Srpska. *Healthcare* 2026, *14*, 2139

**DOI:** 10.3390/healthcare14182890

**Published:** 2026-09-08

**Authors:** Biljana Mijović, Bojan Joksimović, Milena Dubravac Tanasković, Jovan Kulić, Zorica Stojanović, Nataša Stojić, Ljiljana Milošević, Biljana Panin, Galina Ćurčić, Dunja Prokić, Jelena Djekic Malbasa, Predrag Duric

**Affiliations:** 1 Faculty of Medicine Foča, University of East Sarajevo, 73300 Foča, Bosnia and Herzegovina; biljana.mijovic@ues.rs.ba (B.M.); bojannjoksimovic@gmail.com (B.J.); menadub@gmail.com (M.D.T.); zorica.stojanovic@ues.rs.ba (Z.S.); 2 Faculty of Environmental Protection, Educons University, 21208 Sremska Kamenica, Serbia; natasa.stojic@educons.edu.rs (N.S.); ljiljana.curcic@educons.edu.rs (L.M.); biljana.panin@educons.edu.rs (B.P.); galina.curcic@educons.edu.rs (G.Ć.); dunja.prokic@educons.edu.rs (D.P.); predrag.duric@educons.edu.rs (P.D.); 3 Faculty of Medicine, University of Novi Sad, 21137 Novi Sad, Serbia; jelena.djekic-malbasa@mf.uns.ac.rs; 4 Institute for Pulmonary Diseases of Vojvodina, 21204 Sremska Kamenica, Serbia; 5 Faculty of Pharmacy Belgrade, Educons University, 11000 Belgrade, Serbia

**Error in Table**

In the original publication [1], there was a mistake in Table 2 as published. Most of the data in Table 2 were incorrect. The corrected Table 2 appears below.

**Error in Figure**

There was a mistake in Figure 1. The corrected Figure 1 appears below.

**Text Correction**

A correction has been made to the Abstract:

**Background:** The Health in All Policies (HiAP) approach promotes the systematic integration of health considerations into policymaking across non-health sectors to address social determinants of health and improve population health outcomes. Although increasingly recognized internationally, the extent of its legislative implementation in the Republic of Srpska remains unclear. **Objective:** To assess the extent to which HiAP principles are embedded in sectoral legislation in the Republic of Srpska and to identify strengths and gaps in current legal frameworks. **Methods:** A structured content analysis of the text of laws was conducted across ten priority sectors. Twenty-two laws were reviewed using an analytical framework based on five core HiAP components: health outcomes, intersectoral governance, health equity, evidence-informed policymaking, and monitoring and accountability. The presence and distribution of these components were systematically examined across sectors. **Results:** Health considerations were frequently reflected in the text of the analysed legislation. Explicit references to health outcomes were identified in more than 60% of the laws, while references to intersectoral governance were identified in more than 40%. However, integration was uneven across sectors. Health equity principles were inconsistently incorporated, while monitoring, evaluation, and accountability mechanisms were insufficiently developed, with explicit provisions found in fewer than half of the laws. No analysed law formally mandated Health Impact Assessment as a policy instrument. **Conclusions:** The Republic of Srpska has an existing legislative basis for advancing HiAP, but implementation remains fragmented rather than systematic. Addressing identified gaps—particularly in health equity, Health Impact Assessment, and health-oriented monitoring mechanisms—through targeted legislative reforms could improve policy coherence, accountability, and long-term sustainability. To our knowledge, this is the first systematic content analysis of HiAP integration in the sectoral legislation of the Republic of Srpska; it applies a replicable analytical framework derived from the WHO HiAP Framework for Country Action that is transferable to other decentralised and transitional governance settings.

A correction has been made to Section 3.1, Paragraph 1:

A mapping of legal instruments was carried out across the priority sectors identified in the Republic of Srpska. Although the original plan included laws, bylaws, regulations, strategies, and plans, the mapping exercise revealed that framework laws and subordinate legal acts constituted the vast majority of relevant instruments. To maintain consistency and comparability, the analysis was therefore confined to laws. In total, twenty-two sectoral laws were examined, covering energy, agriculture, labour, transport, urban planning, emergency management, water management, environmental protection, education and local governance (Table 1). Most of these laws were adopted between 2006 and 2017. The earliest in the sample is the Law on Agricultural Land and Law on Waters from 2006, while the most recent—the Law on Preschool Education—date from 2025. This distribution reflects a period of considerable legislative activity, with many of the analysed laws having undergone revisions in recent years.

A correction has been made to Section 3.4, Paragraph 1:

Provisions for intersectoral governance appear in 20 laws (90.9%), with explicit mechanisms in 9 laws (40.9%). Formalised governance is most evident in transport, emergency management, and food safety legislation.

A correction has been made to Section 3.5, Paragraph 1:

This component shows the greatest variation. Explicit provisions appear in 9 laws (40.9%), while 5 laws (22.7%) address these issues implicitly, and 8 laws (36.4%) contain no such provisions.

A correction has been made to Section 3.6, Paragraph 1: 

Evidence-based approaches appear in 14 laws (63.6%), with explicit requirements in 11 laws (50.0%), particularly in technical and risk-prone sectors.

A correction has been made to Section 3.7, Paragraph 1:

This component represents the most significant gap. Explicit mechanisms appear in 7 laws (31.8%), implicit monitoring in 6 laws (27.3%), while 9 laws (40.9%) contain no relevant provisions.

**Error in Funding**

A correction has been made to the Funding:

This research is part of the project, entitled “Implementation of the World Health Organization Concept ‘Health in All Policies’: A Comparative Analysis of Achieved Results in Autonomous Province of Vojvodina and the Republic of Srpska”, financially supported by the Provincial Secretariat for Higher Education and Scientific Research of the Autonomous Province of Vojvodina, Republic of Serbia; Research project contract number: 004297127 2025 09418 003 000 000 0001 04 002.

**Reference Correction**

Reference 13 has been replaced with a new reference [2].

The authors state that the scientific conclusions are unaffected. This correction was approved by the Academic Editor. The original publication has also been updated.

## Figures and Tables

**Figure 1:**
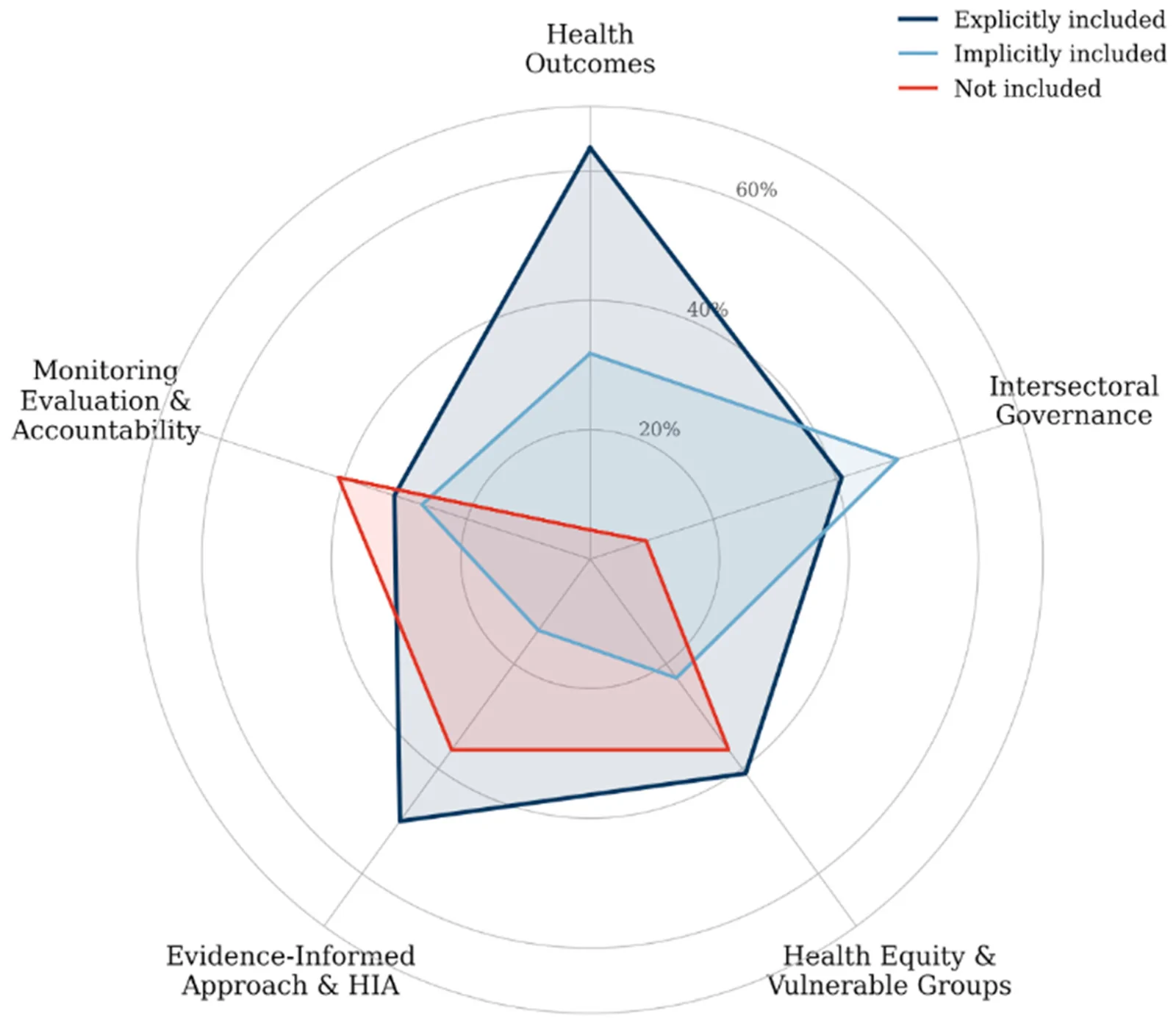
Distribution of HiAP component integration across the 22 analysed laws (percentage of laws coded as explicitly included, implicitly included, and not included for each of the five framework components). Values correspond to Table 2.

**Table 2:** Comprehensive assessment of HiAP component integration across all reviewed laws (*n* = 22).

HiAP Component	Explicitly Included n (%)	Implicitly Included n (%)	Not Included n (%)
1. Consideration of Health Outcomes	14 (63.6%)	7 (31.8%)	1 (4.6%)
2. Intersectoral Governance Mechanisms	9 (40.9%)	11 (50.0%)	2 (9.1%)
3. Health Equity and Vulnerable Groups	9 (40.9%)	5 (22.7%)	8 (36.4%)
4. Evidence-Informed Approach and Health Assessments	11 (50.0%)	3 (13.6%)	8 (36.4%)
5. Monitoring, Evaluation and Accountability for Health	7 (31.8%)	6 (27.3%)	9 (40.9%)
