# Supplementary material for: Health in All Policies in Sectoral Legislation: A Content Analysis of Selected Laws in the Republic of Srpska
Source: Healthcare (Basel). 2026 Jul 16;14(14):2139. doi: 10.3390/healthcare14142139 (PMC13411589; doi:10.3390/healthcare14142139)
Supplement: Supplementary file 1 [file healthcare-14-02139-s001.zip › healthcare-4303812-supplementary.pdf]

Supplementary Table S1. Assessment of Republic of Srpska Legislation Using the Health in All Policies Analytical Framework

Legislative Act	Sector	Health Outcomes	Intersectoral Governance	Vulnerable Groups	Use of Health Evidence	Monitoring, Evaluation and Accountability
Law on Energy	Energy	Implicitly included	Implicitly included	Implicitly included	Implicitly included	Not included
Law on Geological Explorations	Energy	Explicitly included	Implicitly included	Not included	Explicitly included	Explicitly included
Law on Renewable Energy Sources	Energy	Implicitly included	Implicitly included	Not included	Implicitly included	Not included
Law on Mining	Energy	Explicitly included	Implicitly included	Not included	Explicitly included	Implicitly included
Law on Food	Agriculture	Explicitly included	Explicitly included	Explicitly included	Explicitly included	Explicitly included
Law on Agriculture	Agriculture	Implicitly included	Implicitly included	Not included	Not included	Not included
Law on Agricultural Land	Agriculture	Implicitly included	Explicitly included	Implicitly included	Explicitly included	Implicitly included
Law on Labour	Labour	Explicitly included	Not included	Explicitly included	Not included	Not included
Law on Road Traffic Safety	Transport	Explicitly included	Explicitly included	Explicitly included	Explicitly included	Explicitly included
Law on Road Transport of Passengers and Goods	Transport	Explicitly included	Explicitly included	Implicitly included	Explicitly included	Explicitly included
Law on Railways	Transport	Explicitly included	Explicitly included	Implicitly included	Explicitly included	Explicitly included

Law on Spatial Planning and Construction	Urban Planning	Implicitly included	Implicitly included	Implicitly included	Not included	Not included
Law on Protection and Rescue in Emergency Situations	Emergency Management	Explicitly included	Explicitly included	Explicitly included	Explicitly included	Implicitly included
Law on Waters	Water Management	Implicitly included	Implicitly included	Not included	Implicitly included	Implicitly included
Law on Waste Management	Environment	Explicitly included	Implicitly included	Not included	Explicitly included	Implicitly included
Law on Air Protection	Environment	Explicitly included	Implicitly included	Explicitly included	Explicitly included	Explicitly included
Law on Environmental Protection	Environment	Explicitly included	Explicitly included	Not included	Explicitly included	Implicitly included
Law on Primary Education	Education	Explicitly included	Implicitly included	Explicitly included	Not included	Not included
Law on Preschool Education	Education	Explicitly included	Explicitly included	Explicitly included	Not included	Explicitly included
Law on Secondary Education	Education	Explicitly included	Implicitly included	Explicitly included	Not included	Not included
Law on Higher Education	Education	Not included	Not included	Not included	Not included	Not included
Law on Local Self-Government	Local Governance	Implicitly included	Explicitly included	Explicitly included	Not included	Not included
